# Red blotch disease alters grape berry development and metabolism by interfering with the transcriptional and hormonal regulation of ripening

**DOI:** 10.1093/jxb/erw506

**Published:** 2017-01-27

**Authors:** Barbara Blanco-Ulate, Helene Hopfer, Rosa Figueroa-Balderas, Zirou Ye, Rosa M. Rivero, Alfonso Albacete, Francisco Pérez-Alfocea, Renata Koyama, Michael M. Anderson, Rhonda J. Smith, Susan E. Ebeler, Dario Cantu

**Affiliations:** 1 Department of Viticulture and Enology, University of California Davis, Davis, CA 95616, USA; 2 Department of Plant Sciences, University of California Davis, Davis, CA 95616, USA; 3 Department of Food Science, The Pennsylvania State University, University Park, PA 16802, USA; 4 CEBAS-CSIC, Campus de Espinardo, 30100, Murcia, Spain; 5 Department of Agronomy, Londrina State University, Celso Garcia Cid Road, Londrina, PR, 86057-970, Brazil; 6 University of California Cooperative Extension, Sonoma County, Santa Rosa, CA 95403, USA

**Keywords:** Developmental regulation, metabolic flux, perennial woody crop, plant–virus interaction, secondary metabolism, véraison, viral disease

## Abstract

*Grapevine red blotch-associated virus* (GRBaV) is a major threat to the wine industry in the USA. GRBaV infections (aka red blotch disease) compromise crop yield and berry chemical composition, affecting the flavor and aroma properties of must and wine. In this study, we combined genome-wide transcriptional profiling with targeted metabolite analyses and biochemical assays to characterize the impact of the disease on red-skinned berry ripening and metabolism. Using naturally infected berries collected from two vineyards, we were able to identify consistent berry responses to GRBaV across different environmental and cultural conditions. Specific alterations of both primary and secondary metabolism occurred in GRBaV-infected berries during ripening. Notably, GRBaV infections of post-véraison berries resulted in the induction of primary metabolic pathways normally associated with early berry development (e.g. thylakoid electron transfer and the Calvin cycle), while inhibiting ripening-associated pathways, such as a reduced metabolic flux in the central and peripheral phenylpropanoid pathways. We show that this metabolic reprogramming correlates with perturbations at multiple regulatory levels of berry development. Red blotch caused the abnormal expression of transcription factors (e.g. NACs, MYBs, and AP2-ERFs) and elements of the post-transcriptional machinery that function during red-skinned berry ripening. Abscisic acid, ethylene, and auxin pathways, which control both the initiation of ripening and stress responses, were also compromised. We conclude that GRBaV infections disrupt normal berry development and stress responses by altering transcription factors and hormone networks, which result in the inhibition of ripening pathways involved in the generation of color, flavor, and aroma compounds.

## Highlight

Grapevine red blotch interferes with the hormonal and transcriptional regulation of berry development, leading to suppression of secondary metabolism and induction of photosynthesis at late ripening stages.

## Introduction

Viruses are obligate intracellular pathogens that require living host cells to replicate and spread in the infected plant. During compatible interactions, viruses overcome the plant immune system and hijack host cellular processes to establish active infections (Nelson and Citovsky, 2005). Viruses disrupt the plant cell cycle, inhibit cell death pathways, restrict macromolecular trafficking, alter cell signaling, protein turnover, and transcriptional regulation, and suppress defense mechanisms. The interference with these processes in the host leads to a wide range of plant developmental and physiological defects (Laliberté and Sanfaçon, 2010; Pallas and García, 2011; Hanley-Bowdoin *et al.*, 2013).

Cultivated grapevines (*Vitis vinifera* L.) are highly susceptible to a variety of viruses and viroids, which cause significant crop losses and shorten the productive life of vineyards. More than 65 different viral species classified in at least 15 families have been reported to infect grapevines, which represents the highest number of viruses so far detected in a single cultivated plant species (Martelli, 2014). Although these viruses are generally transmitted by plant-feeding insects or soil-borne nematodes, they can also be spread through infected propagation material (Maliogka *et al.*, 2015).

Grapevine red blotch is a viral disease discovered in northern California in 2008 that has become a major economic problem for the wine industry in the USA (Sudarshana *et al.*, 2015). This disease is caused by the *Grapevine red blotch-associated virus* (GRBaV), a circular ssDNA virus with resemblance to geminiviruses, which infects wine grape cultivars with significant detrimental effects on productivity (Krenz *et al.*, 2012, 2014; Poojari *et al.*, 2013; Seguin *et al.*, 2014; Al Rwahnih *et al.*, 2015). The incidence and severity of the red blotch symptoms vary depending on the grape cultivar, environmental conditions, and cultural practices (Sudarshana *et al.*, 2015). In red-skinned varieties, GRBaV infections result in the appearance of red patches on the leaf blades, veins, and petioles; in white-skinned varieties, they manifest as irregular chlorotic regions on the leaf blades. GRBaV also affects berry physiology, causing uneven ripening, higher titratable acidity, and lower sugar and anthocyanin content, among others (Sudarshana *et al.*, 2015). Consequently, must and wine produced from infected berries present altered flavor and aroma.

To date, there is limited information on how GRBaV infections affect grape metabolism. Comprehensive analyses to study specific cellular processes that GRBaV exploits to promote infections in berries are still needed, in particular those that relate to changes in berry chemical composition during fruit development. Grape berry development exhibits a double sigmoid growth pattern with three distinct phases: early fruit development, lag phase, and berry ripening. Most metabolic pathways that promote desired quality traits in grape berries are induced during ripening. The onset of ripening (i.e. véraison) is accompanied by significant changes in berry physiology and metabolism, including softening, sugar accumulation, decrease in organic acids, and synthesis of anthocyanins and other secondary metabolites that define the sensory properties of the fruit (Conde *et al.*, 2007). Berry ripening is controlled by multiple regulatory pathways, and occurs in an organized and developmentally timed manner. Interactions between transcriptional regulators and plant hormones regulate the initiation and progression of ripening processes (Karlova *et al.*, 2014). Like other non-climacteric fruit, grape berries do not display a strong induction of ethylene production and respiration rate at véraison, and the activation of ripening events does not depend primarily on ethylene signaling. Even though the hormonal control of grape berry development is not completely understood, it is established that abscisic acid (ABA), brassinosteroids, and ethylene are positive regulators of ripening processes, while auxin delays the initiation of ripening (Davies *et al.*, 2012; Kuhn *et al.*, 2014). In the context of virus–grape berry interactions, dissecting the mechanisms that regulate ripening and plant defenses may provide new opportunities to develop vineyard management strategies to control viral diseases and ameliorate the negative effects on berry quality.

In this study, we integrated genome-wide transcriptional profiling, targeted chemical and biochemical analyses, and demonstrated that grapevine red blotch disrupts ripening and metabolism of red-skinned berries. We sampled berries at different ripening stages from vines infected with GRBaV and healthy vines in two vineyards. We identified grape metabolic pathways that were altered in ripening berries because of the viral infection. We determined that GRBaV-induced pathways that are normally associated with early fruit development in berries at late stages of ripening, and suppressed secondary metabolic pathways that occur during normal berry ripening and/or in response to stress. Using targeted metabolite profiling and enzyme activity analyses, we confirmed the impact of GRBaV on phenylpropanoid metabolism. We identified specific ripening-related processes that were disturbed in GRBaV-infected berries. Remarkably, these processes included alterations in ripening regulatory networks mediated by transcriptional factors, post-transcriptional control, and plant hormones, which lead to berry developmental defects caused by red blotch.

## Materials and methods

### Biological material

Grape (*Vitis vinifera* cv. Zinfandel) berries were collected in 2015 from the Oakville Experimental Vineyard (Viticulture and Enology Department, UC Davis, Oakville, CA, USA; Oakville, henceforth) and a commercial vineyard in Healdsburg (Sonoma, CA, USA; Healdsburg, henceforth). Sampling was performed between 08:00 h and 11:00 h. Location and weather information for the days of collection are provided in Supplementary Fig. S1 and Supplementary Table S1 at *JXB* online, respectively. Before the first berry sampling, grapevines were pre-screened by a diagnostic laboratory (AgriAnalysis LLC) for the presence of GRBaV and other common grapevine viruses (i.e. *Grapevine leafroll-associated virus* type 1–9, *Grapevine virus A*, *Grapevine virus B*, *Grapevine virus D*, and *Grapevine fanleaf virus*). Vines that tested positive only for GRBaV were included in the study. Vines negative for all tested viruses and that did not show symptoms of viral disease were used as healthy controls. GRBaV infections in the berries were further validated in all samples, including controls, as described below. In Oakville, berries were collected from three GRBaV-positive vines and six control vines. In Healdsburg, six GRBaV-positive vines and three control vines were sampled. Each biological replicate was composed of a pool of at least 20 berries collected from a single vine (i.e. nine biological replications from each vineyard, representing GRBaV-positive vines and controls). Individual berries were obtained at four phenological stages based on the modified Eichhorn–Lorenz (E-L) system (Coombe, 1995): (i) pre-véraison that corresponded to green berries beginning to soften (E-L 34); (ii) véraison that referred to softer berries with 50% red color development (E-L 35); (iii) post-véraison including soft and 100% red-colored berries (E-L 37); and (iv) harvest represented by fully ripe berries equivalent to those used for commercial harvest (E-L 38). On the day of sampling, berries were deseeded, frozen in liquid nitrogen, and ground to powder (skin and pulp). Brix measurements were performed using the ground tissue powder. Total anthocyanin content was measured using 100 mg of grape ground tissue suspended in 350 µl of acidified 1% (v/v) methanol as described in Blanco-Ulate *et al.* (2015).

### Nucleic acid extraction

Sixty-nine samples representing all the biological replicates from GRBaV-positive and control samples at four ripening stages obtained in the two vineyards were used for RNA and DNA extraction. Total RNA was obtained from 2 g of ground tissue as described in Blanco-Ulate *et al.* (2013). DNA was isolated from 1 g of ground grape tissue as in Chin *et al.* (2016) with the addition of proteinase K (400 µg g^−1^ of berry tissue) to the extraction buffer.

### mRNA sequencing and analysis

Sequencing of mRNA was performed with three biological replicates of GRBaV-positive and control berries at each ripening stage and from both locations. The RNA samples not sequenced were used for RT-qPCR validation. Libraries were prepared following the TruSeq RNA Sample preparation kit v.2 (Illumina) and sequenced (single-end, 50 bp) on an Illumina HiSeq3000 (DNA Technologies Core, UC Davis). Sequences are available in GEO (accession: GSE85812). Reads were processed and mapped as described in Blanco-Ulate *et al.* (2015). Sequences of the six GRBaV genes (YP_008400113.1–YP_008400118.1) were combined with the transcriptome of *V. vinifera* var. PN40024 (version V1) and used as a reference for mapping. Normalization and differential expression testing were performed using DESeq2 (Love *et al.*, 2014). We followed the VitisNet functional annotations (Grimplet *et al.*, 2009). Functional enrichment was computed using Fisher’s exact test (*P*≤0.05; genes in enriched category ≥2).

### Quantitative PCR (qPCR)

We used qPCR to determine the presence of viral particles in the berries using DNA extracted from all 69 samples and primers specific to the GRBaV genome sequence (Supplementary Table S2); leaves from certified virus-free grapevines were included as additional negative controls (Foundation Plant Services, UC Davis). For relative gene expression analyses, RNA from all the biological replicates mentioned in the nucleic acid extraction section was reverse-transcribed to cDNA with a M-MLV Reverse Transcriptase (Promega). qPCRs were performed on a QuantStudio3 using SYBR Green Master Mix (Applied Biosystems) with the following conditions: 95 °C (10 min), followed by 40 cycles of 95 °C (3 s) and 60 °C (30 s). Primer efficiency was confirmed to be >90% using triplicate DNA or cDNA dilutions (0, 1:1, 1:4, 1:16, 1:64, and 1:256). Primer specificity was checked by analyzing the melting curves at temperatures between 60 °C and 95 °C. Viral DNA and grape transcript levels are presented as linearized relative-to-reference expression values using the formula 2^(Reference gene CT–Viral DNA region or Grape gene of interest CT)^. Three references genes were tested: *VIT_02s0012g00910*, *VIT_18s0001g00360*, and *VIT_04s0044g00580* (*VvActin*). The latter was chosen as the main reference gene based on correlation analyses with the RNAseq data (Supplementary Fig. S2) and its known expression stability in ripening grape berries (Licausi *et al.*, 2010; Blanco-Ulate *et al.*, 2015.

### Targeted metabolite profiling

Targeted metabolite profiling was performed on all 69 samples. Samples were prepared by diluting 500 mg of ground tissue into 1 ml of 1% (v/v) high purity formic acid (Amresco) in LC-MS grade water (J.T. Baker). Chemical standards were sinapic acid, ferulic acid, *p*-coumaric acid, shikimic acid, gallic acid, naringenin, epicatechin, catechin, gallocatechin, quercetin, quercetin-3-*O*-rutinoside (rutin), quercetin-3-*O*-glucoside (Sigma-Aldrich), kaempferol (Alfa Aesar), and resveratrol (MP Biochemicals). The anthocyanin standards were malvidin-3-*O*-glucoside, cyanidin-3-*O*-glucoside, delphinidin-3-*O*-glucoside, pelargonidin-3-*O*-glucoside, and petunidin-3-*O*-glucoside (Extrasynthese).

Analyses were carried out on an Agilent 1290 UHPLC system with thermostated column compartment (37 °C), temperature-controlled autosampler (4 °C), and diode array detector (DAD), coupled to an Agilent 6460 tandem mass spectrometer. For chromatographic separation, we used a Kinetex PFP guard (4.6 × 2.0 mm) and an analytical column (4.6 × 100 mm, 100 Å, particle size 2.6 μm; Phenomenex). A 7 μl aliquot of the sample was injected for LC-DAD-MS/MS analysis. The mobile phases were 1% (v/v) formic acid in water (solvent A) and 1% (v/v) formic acid in acetonitrile (solvent B) with a flow rate of 0.7 ml min^−1^. The gradient was adapted from de Ferrars *et al.* (2014) as follows: 0–7 min, solvent B (1–7.5%); 7–14 min, solvent B (7.5–7.6%); 14–17 min, solvent B (7.6–10%); 17–18.5 min, solvent B (10–12%); 18.5–20 min, solvent B (12–12.5%); 20–24 min, solvent B (12.5–30%); 24–26 min, solvent B (30%); 26–30 min, solvent B (30–90%); 30–34 min, solvent B (90–99%); 34–38 min, solvent B (99–1%). The sample was first detected using a DAD, which acquired data every 2 s from 190 nm to 600 nm with a resolution of 2.0 nm, and subsequently by an Agilent 6460 tandem mass spectrometer (multiple reaction monitoring mode; ≥3 spectra s^–1^ for each transition). An Agilent Jetstream ESI source (positive or negative ion mode) was used for all MS/MS analyses. The drying gas was operated at 8 l min^−1^ and 300 °C, and sheath gas was set at 11 l min^−1^ and 350 °C. The nebulizer pressure was 40 psi. Capillary voltage was set to 4000 V and 3500 V for positive and negative mode, respectively. The nozzle voltage was 500 V for both modes.

The LC-DAD-MS/MS data were acquired using MassHunter (B.06.00, Agilent), and processed and visualized with MassHunter Qualitative and Quantitative Analysis (B.07.00, Agilent). Method detection limits for all compounds were determined as the amount of the analyte that gives a signal statistically greater than the population mean value of zero, using the second lowest calibration point. Five-point calibration curves were established for each compound, covering the range of observed concentrations, by injecting standard compounds in duplicate. Sample concentrations were expressed on a fresh weight basis. To account for day-to-day variability, a quality control (QC) sample was prepared by mixing nine samples of GRBaV-positive and control berries at different ripening stages. The same QC sample was extracted in the same way as the samples on each day, and used for sample concentration normalization. The abundance of all metabolites was expressed as ng g^–1^ FW.

### Enzymatic assays

Measurements of enzyme activity were performed with three biological replicates of GRBaV-positive and control berries from the Oakville vineyard at three ripening stages: véraison, post-véraison, and harvest. The extractions for the enzymes: phenylalanine ammonia-lyase (PAL; EC 4.1.3.5), *trans*-cinnamate 4-monooxygenase (C4H; EC 1.14.13.11), chalcone synthase (CHS; EC 2.3.1.74), flavonone 3-hydroxylase (F3H; EC 1.14.11.9), dihydroflavonol 4-reductase (DFR; EC 1.1.1.219), flavonol synthase (FLS; EC 1.14.11.23), and UDP-glucose:flavonoid 3-*O*-glucosyltransferase (UF3GT; EC 2.4.1.115) were performed as in Blanco-Ulate *et al.* (2015), with the exception that 50 mg of freeze-lyophilized grape tissue per sample were used instead of fresh tissue. Stilbene synthase (STS; EC 2.3.1.95) activity was assayed as in Kodan *et al.* (2002) and Camont *et al.* (2009) with modifications: freeze-lyophilized samples (50 mg) were homogenized in 1 ml of 100 mM HEPES-NaOH pH 7.0 containing 3 mM DTT, 20% (v/v) glycerol, 1% (w/v) BSA, and 1% (w/v) polyvinylpolypyrrolidone (PVPP). Homogenates were passed through Miracloth (Merk Millipore), transferred to 1.5 ml tubes, and centrifuged at 8000 *g* for 30 min at 4 ºC. The supernatants were transferred to new 1.5 ml tubes and centrifuged at 12 000 *g* for 15 min at 4 ºC. Supernatants were passed through a pre-buffered NICK spin column (Sephadex G50 DNA grade). STS activity was assayed in a reaction mixture with 50 µl of grape extracts, 100 µl of 20 mM HEPES-NaOH pH 7.5 containing 2 mM β-mercaptoethanol, 5 mM EDTA, and 0.03 mM DTT. The reaction was started by the addition of 50 µl of a mix containing 15 µM malonyl-CoA and 20 µM cinnamoyl-CoA. Changes in the absorbance at 290 nm due to the formation of *cis*- and *trans*-resveratrol were measured during 10 min. Protein concentrations were measured as in Bradford (1976).

### Phytohormone analyses

Hormone abundances were obtained from three biological replicates of GRBaV-positive and control berries from Oakville at four ripening stages. The abundances (μg g^–1^ DW) of ABA, gibberellic acid 3 (GA_3_), the active cytokinin *trans*-zeatin (tZ), and salicylic acid (SA) were analyzed as described in Albacete *et al.* (2008) with some modifications. Briefly, 0.1 g of freeze-lyophilized samples were homogenized and extracted using 1 ml of cold extraction mixture of methanol/water (80/20, v/v; –20 °C). Samples were analyzed using a UHPLC-MS system consisting of an Accela Series UHPLC coupled to an Exactive mass spectrometer (ThermoFisher Scientific) with a heated electrospray ionization (HESI) interface. Mass spectra were obtained using Xcalibur 2.2 (ThermoFisher Scientific). For quantification, calibration curves were constructed for each phytohormone (1, 10, 50, and 100 µg l^–1^) and corrected for 10 µg l^–1^ deuterated internal standards. Recovery percentages ranged from 92% to 95%.

## Results

### Sampling of naturally GRBaV-infected grape berries in multiple vineyards

To determine the impact of grapevine red blotch on berry physiology, we studied naturally occurring GRBaV infections in distinct wine grape-growing regions in northern California (USA). We sampled red-skinned grape berries (*V. vinifera* cv. Zinfandel) from two different vineyards, one in Oakville (Napa County) and one in Healdsburg (Sonoma County; Supplementary Fig. S1; Supplementary Table S1). We used multiple vineyard sites to focus on observations consistently made across environments and, thus, to exclude factors associated with specific environmental or cultural conditions.

**Fig. 1.**
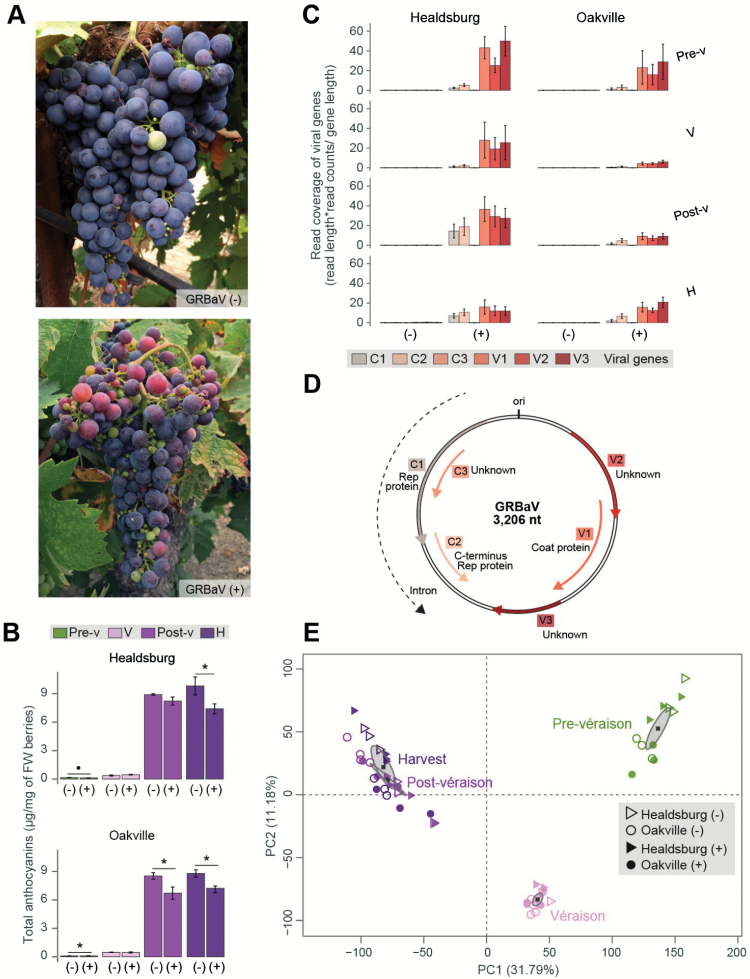
Naturally occurring GRBaV infections in a red-skinned grapevine cultivar. (A) Berry cluster asynchronous development observed in grapevines that tested positive for the presence of GRBaV. (B) Total anthocyanins from GRBaV-positive and GRBaV-negative berries collected at four specific ripening stages. Symbols indicate significant fold changes (•*P*≤0.1, *P≤0.05). (C) Expression of GBRaV genes in berries from GRBaV-positive (+) or negative (–) control vines across four ripening stages represented as mapped read coverage based on RNAseq data. (D) Genome organization of GRBaV obtained from Krenz *et al.* (2014). Six ORFs are denoted with colored arrows and their gene names are included. The origin of replication (ori) is also depicted. (E) Principal component analysis of GRBaV-positive and negative berries at four ripening stages and collected from two independent vineyards. The quantitative variables correspond to transcript abundance of 25 995 grape genes. Each circle or triangle represents a biological replicate. Gray ellipses define confidence areas (95%) for each stage, while gray squares represent their corresponding centers of gravity. Pre-v, pre-véraison; V, véraison; Post-v, post-véraison; H, harvest; PC, principal component. Error bars correspond to SEs.

Prior to sampling, vines were screened for the presence of GRBaV and other common grapevine viruses. The appearance of red blotch symptoms on leaves of GRBaV-positive vines and not on those of healthy controls confirmed the initial viral testing. We sampled grape berries from vines that tested positive for GRBaV and negative for other common grapevine viruses. At the same time, we also collected berries from vines that tested negative for all viruses and included them in the study as healthy controls. In order to determine the impact of the disease on berry development and metabolism, we collected GRBaV-positive and control berries at comparable developmental stages: pre-véraison (E-L 34), véraison (E-L 35), post-véraison (E-L 37), and harvest (E-L 38). This sampling strategy also aimed to limit confounding effects due to differences in the progression of ripening between berry clusters of GRBaV-positive and healthy vines. In some cases, we observed that GRBaV-positive vines presented grape clusters with evident uneven ripening (Fig. 1A). Comparisons between berries from GRBaV-positive vines and healthy controls indicated that, at equivalent stages of development, berries affected by red blotch had reduced soluble solids and total anthocyanins (Fig. 1B; Supplementary Table S3) in agreement with previous reports on red-skinned wine grapes (Sudarshana *et al.*, 2015).

Sampled berries were used for genome-wide transcriptional profiling (RNAseq) of viral and grape genes. RNAseq was performed using 3–4 biological replicates of each ripening stage, infection status, and vineyard. We first confirmed the presence of the virus in the berries of GRBaV-positive vines by qPCR amplification of viral DNA (Supplementary Fig. S3). Viral activity in the berries was also assessed by quantifying plant-derived mRNA transcripts of GRBaV genes in the RNAseq data. Plant expression of five out of the six predicted genes in the GRBaV genome (Krenz *et al.*, 2014) was detected in all berry samples obtained from GRBaV-positive vines but not in berries collected from the control vines (Fig. 1C, D). The most expressed GRBaV genes in the berries corresponded to V1, which encodes a coat protein, and V3 with unknown function. Expression levels of the GRBaV genes appeared to change as berries ripened. However, we could not determine to what extent the progression of ripening or other environmental factors influenced the plant’s transcription of viral genes because their pattern of variation between ripening stages differed in the two vineyards (Fig. 1C).

Expression of 25 994 grape genes (86.73% of total annotated genes) was detected by RNAseq across all berry samples. Principal component analysis (PCA) was carried out with the normalized read counts of all detected genes. The two major PCs, which together accounted for 42.97% of the total variability, clearly separated the samples based on ripening stage, regardless of the vineyard of origin or their infection status (Fig. 1E). These results indicated that (i) the intervineyard variation was smaller than the ripening effect, and (ii) the overall progression of ripening was similar between berries from GRBaV-positive and control vines. Therefore, we hypothesized that GRBaV infections in berries have altered the expression of particular grape genes and/or molecular pathways, which could subsequently have led to developmental and metabolic defects.

### Grapevine red blotch alters berry primary and secondary metabolism as well as responses to stress

While the PCA described above (Fig. 1E) indicated that overall transcriptome dynamics associated with berry ripening were not perturbed by the infection, the lower levels of soluble solids and anthocyanins in GRBaV-positive berries, particularly later in development, suggested that red blotch may affect specific primary and secondary metabolic processes. We therefore focused the RNAseq analyses to identify grape molecular pathways that were differentially regulated as a result of GRBaV infections. We identified grape genes with significant differential expression (*P*≤0.05) due to red blotch by comparing GRBaV-positive and GRBaV-negative berries at each ripening stage and independently for each vineyard. We then looked at the intersection of differentially expressed (DE) genes between the two vineyards to identify common responses to red blotch. A total of 932 grape DE genes were found to be consistently down- or up-regulated in infected berries in both vineyards at a given ripening stage, and were classified as GRBaV-responsive genes (115 genes at pre-véraison, 354 at véraison, 309 at post-véraison, and 326 at harvest; Supplementary Table S5). On average these GRBaV-responsive genes showed 0.49 ± 0.22-fold changes (i.e. up- and down-regulations) compared with the healthy controls. Comparing berries at similar ripening stages may have contributed to exclude more dramatic changes in gene expression associated with more pronounced ripening delay due to GRBaV.

**Fig. 2.**
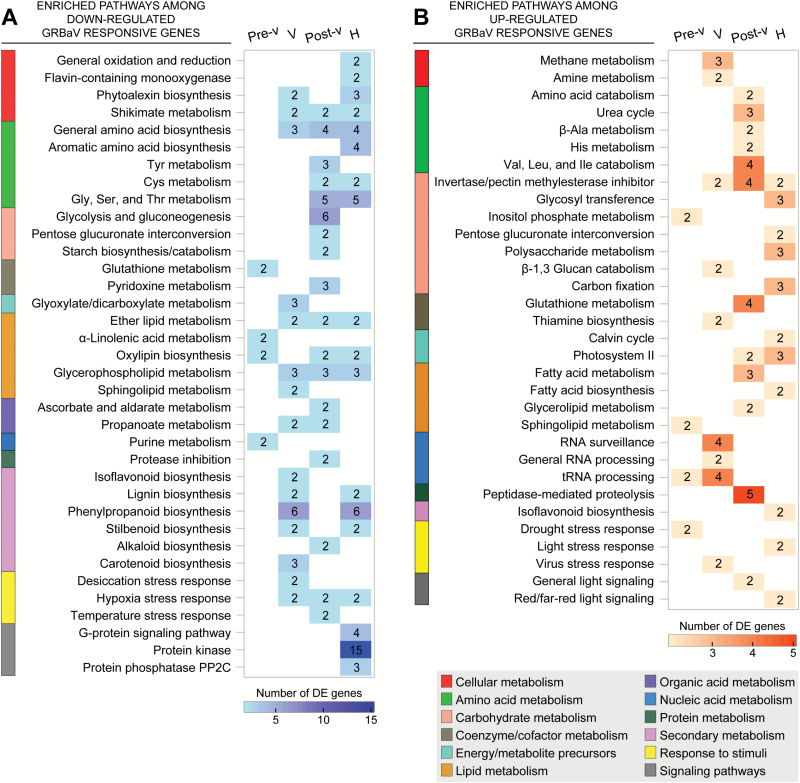
Grape metabolic pathways suppressed or activated in response to GRBaV infections of Zinfandel berries at four ripening stages. The graph depicts enriched (*P*≤0.05) pathways among significantly (*P*≤0.05) down-regulated (A) and up-regulated (B) grape genes in response to GRBaV infections of ripening berries from two vineyards. Color intensities in the heat map correlate with the number of differentially expressed (DE) genes in each pathway. Blank spaces represent pathways that were not significantly enriched at a particular stage or that did not include DE genes. Pre-v, pre-véraison; V, véraison; Post-v, post-véraison; H, harvest.

Key metabolic processes that were suppressed or induced as a consequence of red blotch in ripening berries were identified by enrichment analyses (*P*≤0.05) of the functional categories defined by Grimplet *et al.* (2009) in the set of GRBaV-responsive genes (Fig. 2; Supplementary Table S6). GRBaV infections altered the transcription of several primary metabolic pathways. Amino acid biosynthetic pathways were repressed in GRBaV-positive berries, while amino acid catabolic pathways were induced. Changes in carbohydrate metabolism were also observed; in particular, genes involved in glycolysis/gluconeogenesis and starch metabolism had reduced expression in GRBaV-infected berries. The suppression of these pathways may partially explain the lower soluble solids in the GRBaV-positive berries. Remarkably, GRBaV infections also led to the activation in berries at late stages of ripening of processes that are normally associated with early fruit development, such as photosynthesis-related pathways: both thylakoid electron transfer (PSII proteins: *VIT_12s0028g01080*, *VIT_00s0207g00210*, and *VIT_11s0016g01400*) and the Calvin cycle (ribulose-1,5-bisphosphate carboxylase/oxygenase: *VIT_17s0000g03690*, and fructose-bisphosphate aldolase: *VIT_03s0038g00670*), which are normally suppressed during berry ripening were up-regulated at post-véraison and harvest (Pilati *et al*., 2007). We also detected enrichment of invertase/pectin methylesterase (PME) inhibitors among the up-regulated genes in GRBaV-infected berries (*VIT_02s0012g00500*, *VIT_11s0016g00590*, *VIT_15s0021g00540*, and *VIT_16s0022g00960*). These inhibitors have been reported to control PME activity at early phases of berry development and appear to function as regulators of berry enlargement and softening (Lionetti *et al.*, 2015).

Genes involved in nucleic acid metabolism, including RNA processing and surveillance, showed higher expression in GRBaV-infected berries. These pathways coincided at véraison with the induction of genes involved in stress responses to virus (Fig. 2B; Supplementary Table S6). RNA metabolic pathways are commonly altered in plants during viral infections and have been related to resistance or susceptibility depending on the particular plant–virus interaction (Culver and Padmanabhan, 2007). Red blotch also impacted the transcription of several abiotic stress response pathways. In particular, berries after véraison showed lower expression of genes encoding hypoxia-responsive proteins and heat stress transcription factors, among others (Supplementary Table S6).

A prominent feature of the GRBaV-positive berries was the transcriptional suppression of secondary metabolic pathways, in particular the biosynthesis of phenylpropanoids, stilbenoids, and lignin (Fig. 2A; Supplementary Table S6). Because the lower anthocyanin content observed in the GRBaV-positive berries may have resulted from reduced metabolic flux in the core phenylpropanoid pathway and alterations in flavonoid and anthocyanin biosynthesis, we pursued a deeper evaluation of these pathways using an integrated approach of transcriptional and metabolite profiling coupled to enzymatic analyses.

### Grapevine red blotch suppresses phenylpropanoid metabolism during berry ripening

Twenty-five genes involved in phenylpropanoid metabolism were among the GRBaV-responsive genes and 17 (68%) of them had lower expression levels at particular stages due to the viral infection (Table 1). We also noted that genes with higher expression due to GBRaV infections were mainly involved in isoflavonoid metabolism. We further validated the expression patterns of several of these genes by qRT-PCR (*r*=0.72, *P*=1.41 × 10^–5^; Supplementary Fig. S2) in the GRBaV-positive and control berries.

**Table 1.** Genes from the phenylpropanoid metabolic pathways whose expression was significantly altered by GRBaV infections in both vineyards Functional annotations and gene accessions are provided, in addition to their fold changes in expression when comparing GRBaV-positive (+) versus negative (–) berries. All reported changes correspond to significant up- or down-regulation (**P*≤*0.05).

Gene	Functional annotation	Expression log_2_ fold changes, GRBaV(+)/GRBaV(–)							
		Pre-véraison		Véraison		Post-véraison		Harvest	
		Hld	Okv	Hld	Okv	Hld	Okv	Hld	Okv
*VIT_11s0065g00350*	*Trans*-cinnamate 4-monooxygenase (C4H)					–0.61	–1.14		
*VIT_16s0098g00850*	Caffeic acid 3-*O*-methyltransferase (COMT)			–0.57	–0.40				
*VIT_04s0023g02900*	Ferulate-5-hydroxylase (F5H)							–0.36	–0.86
*VIT_06s0061g00450*	4-Coumaroyl-CoA ligase (4CL)					0.31	0.36		
*VIT_11s0052g01090*	4-Coumaroyl-CoA ligase (4CL)			–1.02	–0.39				
*VIT_12s0035g02070*	Cinnamoyl-CoA reductase (CCR)							0.27	0.53
*VIT_14s0066g01150*	Cinnamoyl-CoA reductase (CCR)			–0.71	–0.31	–0.45	–0.87	–0.50	–0.88
*VIT_02s0012g01570*	Cinnamoyl-CoA reductase (CCR)			–0.26	–0.50			–0.27	–0.29
*VIT_03s0180g00260*	Cinnamyl alcohol dehydrogenase (CAD)					0.30	0.16		
*VIT_18s0117g00550*	Laccase							–0.26	–0.55
*VIT_08s0040g00780*	*P*-Coumaroyl shikimate 3'-hydroxylase isoform							–0.35	–0.39
*VIT_16s0100g00910*	Stilbene synthase (STS)			–0.47	–0.50				
*VIT_16s0100g00900*	Stilbene synthase (STS)							–0.52	–0.72
*VIT_16s0100g01030*	Stilbene synthase (STS)			–0.64	–0.35				
*VIT_16s0100g01000*	Stilbene synthase (STS)							–0.61	–0.73
*VIT_03s0088g00060*	Isoflavone reductase							0.40	0.71
*VIT_03s0088g00140*	Isoflavone reductase							0.53	0.67
*VIT_18s0001g12690*	Isoflavone reductase					0.37	0.50		
*VIT_10s0003g00480*	Orcinol *O*-methyltransferase (OOMT)			–0.42	–0.37				
*VIT_07s0129g00730*	Isoflavone 2'-hydroxylase (I2'H)			0.31	0.28				
*VIT_15s0048g01000*	Dihydroflavanol 4-reductase (DFR)			–0.37	–0.49				
*VIT_12s0034g00130*	UDP-glucose:anthocyanidin 3-*O*-d-glucosyltransferase (UF3GT)	–0.83	–0.60					–0.35	–0.41
*VIT_12s0055g00290*	UDP-glucose:anthocyanidin 3-*O*-d-glucosyltransferase (UF3GT)			0.48	0.52				
*VIT_02s0025g02920*	Quercetin 3-*O*-methyltransferase (OMT)							–0.23	–0.45
*VIT_00s0218g00160*	UDP-rhamnose: rhamnosyltransferase			–0.54	–0.59				

Hld, Healdsburg vineyard; Okv, Oakville vineyard. The complete data set can be accessed in Supplementary Table S5.

**Fig. 3.**
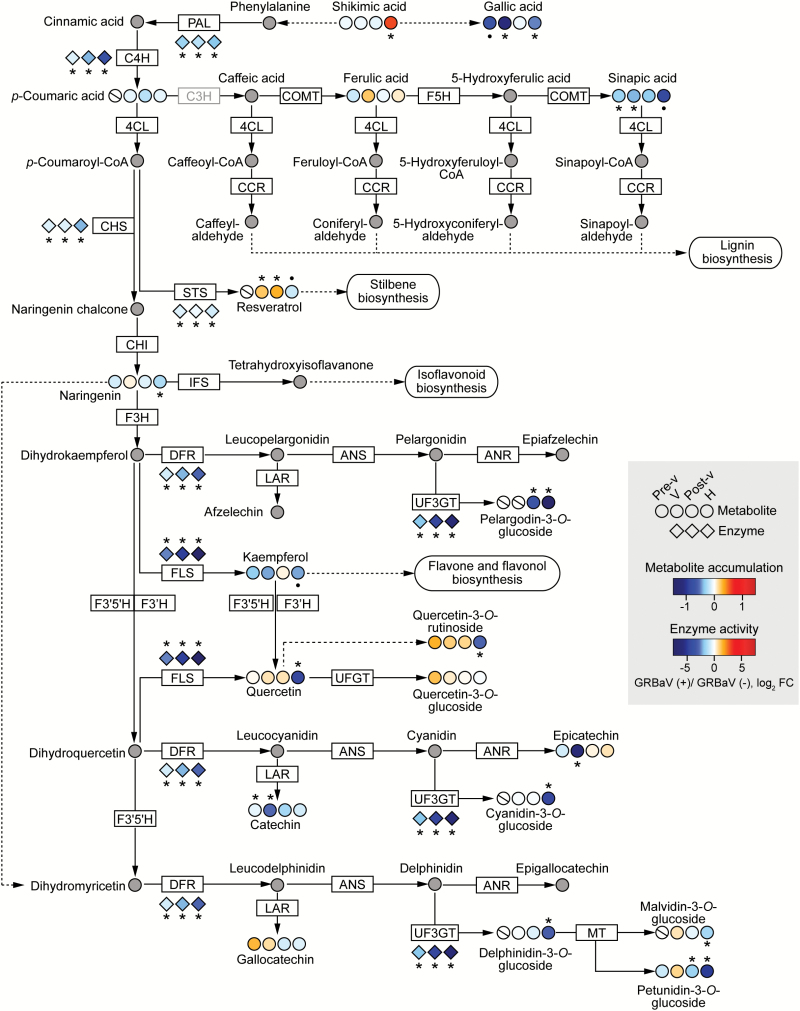
Repression of phenylpropanoid metabolism during GRBaV infections of grape berries. A representation of the central phenylpropanoid and flavonoid pathways based on KEGG pathways (www.genome.jp/kegg/pathway.html, last accessed 4 January 2017). Dashed lines indicate that some steps were omitted. The gray box indicates an enzyme that has not been completely characterized. Circles represent metabolites, and diamonds correspond to enzymes. The colors of the circles and diamonds represent the intensity of fold changes in metabolite accumulation or enzyme activity, respectively, at a given ripening stage when comparing GRBaV-positive versus control berries. Gray circles correspond to metabolites that were not monitored. Symbols indicate significant fold changes (•*P*≤0.1, *P≤0.05). Circles with a cross line refer to metabolites that were not detected in samples of a particular stage. Pre-v, pre-véraison; V, véraison; Post-v, post-véraison; H, harvest; PAL, phenylalanine ammonia-lyase; C4H, *trans*-cinnamate 4-monooxygenase; C3H, p-coumarate 3-hydroxylase; COMT, caffeic acid 3-*O*-methyltransferase; FH5, ferulate-5-hydroxylase; 4CL, 4-coumaroyl-CoA ligase; CCR, cinnamoyl-CoA reductase; CHS, chalcone synthase; STS, stilbene synthase; CHI, chalcone isomerase; IFS, isoflavonoid synthase; F3H, flavonone 3-hydroxylase; DFR, dihydroflavanol 4-reductase; LAR, leucoanthocyanidin reductase; ANS, anthocyanidin synthase; ANR, anthocyanidin reductase; UF3GT, UDP-glucose:anthocyanidin 3-*O*-d-glucosyltransferase; UFGT, UDP-glucose:flavonoid 3-*O*-glucosyltransferase; FLS, flavonol synthase; F3'5'H, flavonoid 3',5'-hydroxylase; F3'H, flavonoid 3'-monooxygenase; MT, methyltransferase.

Most enzymes involved in phenylpropanoid metabolism are encoded by large gene families. There is also high redundancy among these genes, which ensures the functional integrity and plasticity of the phenylpropanoid-related pathways (Vogt, 2010). Therefore, to test the hypothesis that the red blotch-induced transcriptional changes had an actual impact on phenylpropanoid metabolism, we measured the activity of key enzymes and the abundance of compounds involved in these pathways (Supplementary Table S7 and Supplementary Table S8, respectively). We detected significant reductions in activity of seven enzymes that catalyze important steps in the core phenylpropanoid, stilbene, flavonoid, and anthocyanin biosynthetic pathways due to GRBaV infections of berries at three ripening stages (Fig. 3). In addition, the first enzyme committed to flavone and flavonol biosynthesis, flavonol synthase (FLS), had significantly lower activity at post-véraison and harvest stages (Supplementary Table S7). Red blotch altered the accumulation of 17 compounds that result from the phenylpropanoid metabolism and two compounds upstream of this pathway, shikimic acid and gallic acid (Fig. 3; Supplementary Table S8). Most of these compounds showed significantly lower abundance in the GRBaV-positive berries compared with the controls at later stages of ripening. The main anthocyanins present in grape berries: malvidin-3-*O*-glucoside, petunidin-3-*O*-glucoside, delphinidin-3-*O*-glucoside, pelargodin-3-*O*-glucoside, and cyanidin-3-*O*-glucoside, were significantly reduced by red blotch at harvest. Gallic acid, sinapic acid, and quercetin also showed lower abundance in infected berries. Few exceptions to this general suppression of phenolic accumulation were the accumulation of the precursor shikimic acid, which significantly increased in infected berries at harvest, and of resveratrol that showed significantly greater accumulation at véraison and post-véraison. Additional experiments are necessary to understand the accumulation of these two metabolites in the presence of GRBaV: preliminarily, we can hypothesize that the higher abundance of resveratrol is due either to a restriction of subsequent enzymatic steps in stilbene metabolism for which this compound is a substrate, or to the enzymatic hydrolysis of resveratrol glycosides or stilbenoid dimers previously synthesized.

The integrated analysis of transcriptomic, metabolite, and enzyme activity data supported a general repression of the core and peripheral phenylpropanoid pathways, which are normally triggered in red-skinned berries throughout ripening and in response to stress (Conde *et al.*, 2007; Singh *et al.*, 2010; Vogt, 2010). These results suggest that GRBaV infections disrupt secondary metabolic pathways by altering the regulation of berry ripening processes and/or signaling mechanisms related to plant defense. Interestingly, GRBaV infections seemed to have a more a pronounced impact on enzymatic activities and metabolite accumulation than on the expression levels of the genes in the pathway, which in general displayed small fold change differences between healthy and infected samples. This observation further confirms the importance of evaluating metabolic perturbations at multiple regulatory levels.

### Grapevine red blotch perturbs ripening regulatory pathways

**Fig. 4.**
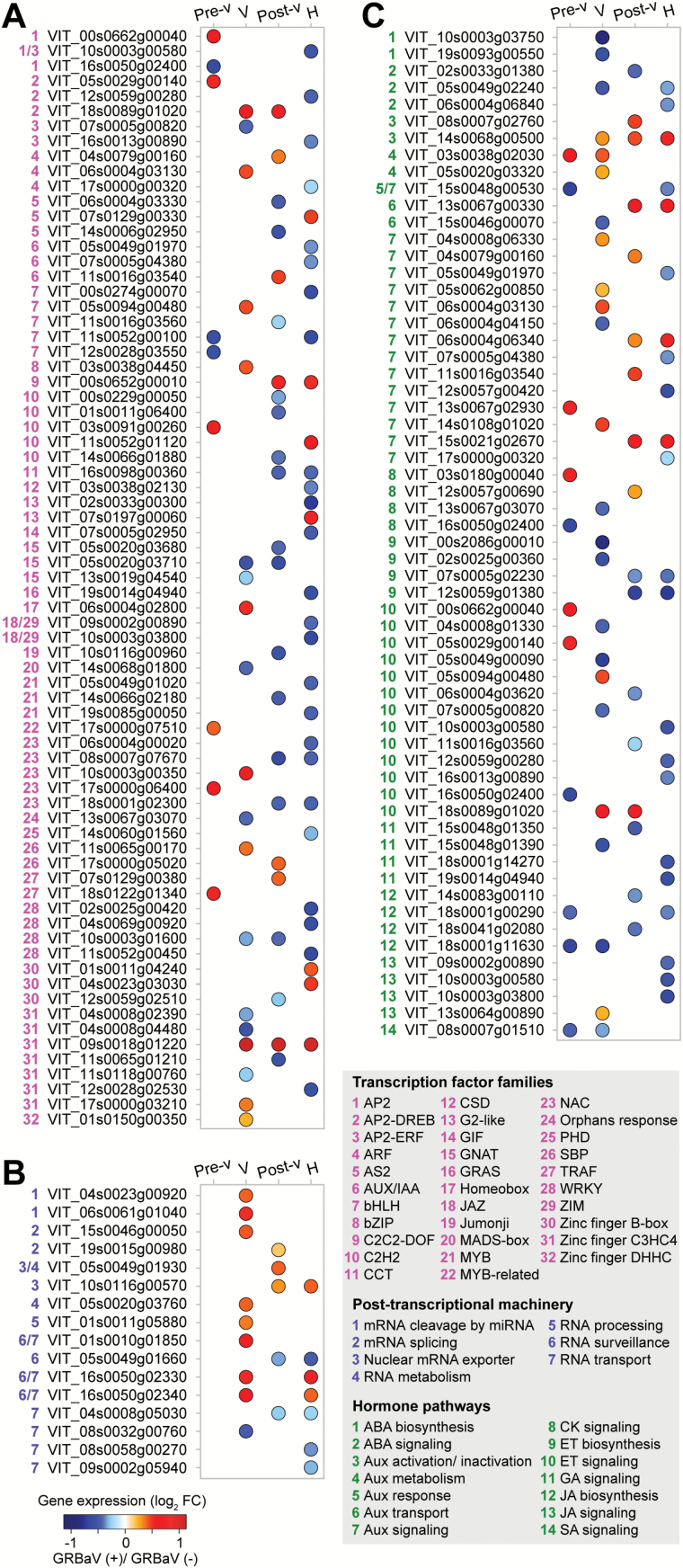
GRBaV infections altered the regulation of berry ripening. Expression changes of ripening-related transcription factors (A), post-transcriptional machinery elements (B), and hormone-related genes (C) as a result of GRBaV infections. The colors of the circles represent the intensity of the expression fold changes (log_2_). The complete data set is available in Supplementary Table S9. Pre-v, pre-véraison; V, véraison; Post-v, post-véraison; H, harvest.

We performed additional analyses of the transcriptomic data to determine the effect of the disease on ripening-related processes. First, we identified 10 663 genes with differential expression during normal ripening of red-skinned berries by pairwise comparisons between the four ripening stages using only the healthy control samples (véraison versus pre-véraison, post-véraison versus véraison, and harvest versus post-véraison; Supplementary Table S9). Out of all these ripening-related genes, 679 were GRBaV-responsive genes. Among the ripening genes altered by GRBaV infections, 128 genes (19.85%) were involved in developmental regulation and signal transduction, from transcriptional and post-transcriptional regulation, to hormone biosynthesis and signaling (Fig. 4; Supplementary Table S9).

Red blotch altered the expression patterns of members of 32 transcription factor families that operate during normal berry ripening (Fig. 4A). For example, three MYB transcription factors (#21 in Fig. 4A) presented a clear down-regulation in ripening berries as a result of red blotch, including *VvMYB15* (*VIT_05s0049g01020*), a main regulator of stilbene biosynthesis. The MYB family has been associated with the control of the phenylpropanoid and peripheral pathways in red-skinned berries (Deluc *et al.*, 2006, 2008; Bogs *et al.*, 2007; Walker *et al.*, 2007; Matus *et al.*, 2008; Höll *et al.*, 2013; Cavallini *et al.*, 2015). Two genes coding for members in the SQUAMOSA Promoter-Binding Protein-Like (SPB) family, *VIT_11s0065g00170* and *VIT_17s0000g05020*, which should be down-regulated during berry ripening (#26 in Fig. 4A), presented higher levels of expression at véraison and post-véraison in infected berries, respectively. SPB transcription factors are involved in the control of developmental transitions of plant tissues (Chen *et al.*, 2010). C3HC4-type RING finger transcription factors also displayed atypical expression profiles during ripening (#31 in Fig. 4A). Although, members of the C3HC4-type RING family have not yet been associated with the control of grape berry ripening, it is known that some regulate plant development and stress responses in Arabidopsis, rice, and tobacco (Von Arnim and Deng, 1993; Ma *et al.*, 2009; Wu *et al.*, 2014). We detected several NAM/ATAF/CUC (NAC) transcription factors with abnormal ripening patterns in the GRBaV-positive berries (#23 in Fig. 4A). NAC transcription factors have been reported to regulate fruit ripening, senescence, and stress responses (Nakashima *et al.*, 2012; Kou *et al.*, 2014; Podzimska-Sroka *et al.*, 2015). Notably, *VvNAC60* (*VIT_08s0007g07670*), which has been reported to be a master regulator of véraison (Palumbo *et al.*, 2014), had lower expression levels in the berries due to red blotch.

Several genes with putative functions in the post-transcriptional control of berry ripening presented altered expression patterns in response to red blotch (Fig. 4B). Among these genes, 70.58% showed higher expression levels in the infected berries, including three homologs of Regulator of nonsense transcripts-1 (UPF1; *VIT_01s0010g01850*, *VIT_16s0050g02330*, and *VIT_16s0050g02340*) that have potential roles in the elimination of nonsense-containing RNAs, a Dicer-like2 protein (*VIT_04s0023g00920*), an Argonaute protein (*VIT_06s0061g01040*), an RNA-dependent RNA polymerase 1 (*VIT_01s0011g05880*), an RNA helicase SDE3-like (*VIT_05s0020g03760*), and a Spliceosome-associated protein (*VIT_15s0046g00050*).

Transcriptional networks of hormones that are known to promote grape berry ripening, ABA and ethylene, were in general inhibited by GRBaV infections (Fig. 4C). Suppression of ABA biosynthesis and signaling genes was detected at véraison and subsequent ripening stages (#1 and #2 in Fig. 4C). Two 9-*cis*-epoxycarotenoid dioxygenases (NCEDs; *VIT_10s0003g03750* and *VIT_19s0093g00550*) that showed significant up-regulation between pre-véraison and véraison in control berries were not induced during ripening of GRBaV-positive berries. Several ethylene biosynthetic genes showed lower expression levels due to red blotch (#9 in Fig. 4C), while genes involved in ethylene signal transduction and responses had altered expression patterns (i.e. up- and down-regulation; #10 in Fig. 4C). The latter included members of the APETALA2/Ethylene Responsive Factors (AP2-ERF), which have been involved in the regulation of plant–pathogen interactions and fruit ripening (Gutterson and Reuber, 2004; Licausi *et al.*, 2013). Moreover, genes involved in gibberellin-mediated signaling had lower expression levels during ripening of infected berries, such as two putative gibberellin receptors (#11 in Fig. 4C).

In contrast, auxin-mediated networks, which prevent ripening processes, showed increased transcriptional levels in response to red blotch (#3 to #7 in Fig. 4C). Twenty-one genes involved in auxin metabolism and signaling had distorted expression patterns in GRBaV-positive berries; 67% of them showed increased levels throughout ripening when compared with the controls. The genes with higher expression levels included: an indole acetic acid (IAA)-amino acid hydrolase (*VIT_08s0007g02760*) that is involved in auxin activation, an auxin influx carrier protein (AUX1-like; *VIT_13s0067g00330*), an Auxin-IAA (AUX-IAA) transcription factor (*VIT_11s0016g03540*), and two Auxin Response Factors (ARFs; *VIT_04s0079g00160* and *VIT_06s0004g03130*).

**Fig. 5.**
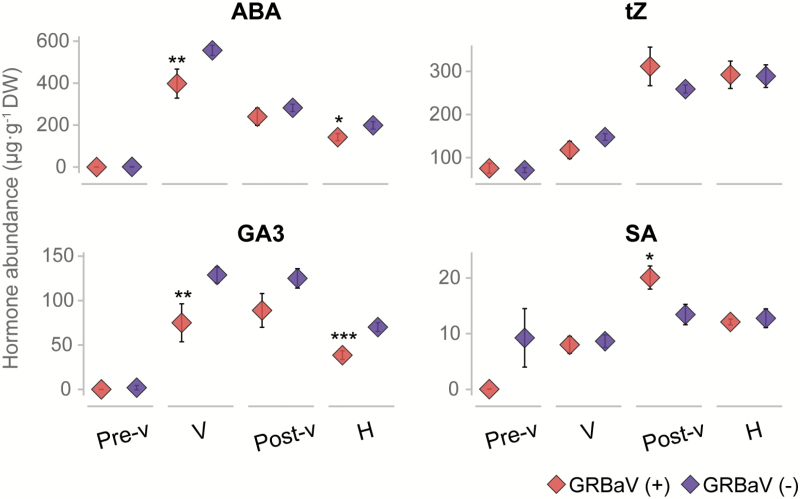
Accumulation of plant hormones in ripening berries as a result of red blotch. Plots show the abundance of specific plant hormones measured in berries collected from the Oakville vineyard. Asterisks indicate significant differences (*P≤0.05, **P≤0.01, ***P≤0.001) when comparing GRBaV-positive samples (+) versus healthy controls (–). ABA, abscisic acid; tZ, *trans*-zeatin, GA3, gibberellic acid 3; SA, salicylic acid. Error bars correspond to SEs. (This figure is available in colour at *JXB* online.)

To provide complementary information to the transcriptional data and help reveal key hormonal imbalances that result from GRBaV infections in ripening berries, we measured the concentrations of ABA, tZ, GA3, and SA (Fig. 5). Although, SA is not yet implicated in ripening processes, it was included as an indicator of plant responses to the virus. The results confirmed that ABA accumulation was significantly lower in GRBaV-positive berries at véraison and harvest stages. A similar pattern was observed for GA3, including significant reductions in abundance due to viral infection. There were no significant differences in the accumulation of tZ when comparing GRBaV-positive and control samples. In the case of SA, the viral infection caused a significant increase in the hormone levels at the post-véraison stage, which may relate to berry mechanisms to cope with the pathogen attack.

Altogether these results demonstrate that GRBaV infections cause reprograming of transcriptional and post-transcriptional regulators in conjunction with hormonal imbalances, leading to the suppression of specific ripening processes and abnormal phenolic composition in red-skinned grape berries.

## Discussion

Understanding how plants respond to external stimuli in the field is crucial to improve agricultural traits under naturally fluctuating conditions. Most studies on plant–pathogen interactions are performed with model organisms in the greenhouse or laboratory, which reduce the confounding effects of the environment, but also challenge the reproducibility of the results in the field (Atkinson and Urwin, 2012; Alexandersson *et al.*, 2014; Suzuki *et al.*, 2014). Compatible plant–virus interactions in perennial woody crops are complex due to the presence of multiple and systemic infections, tissue and developmental stage-specific responses, differences between species and cultivars, and the combination of biotic and abiotic factors during the crop season (Naidu *et al.*, 2014). The application of a system biology approach to study red blotch under multiple vineyard conditions allowed us to explore grapevine responses to GRBaV infections in real agronomic settings and to characterize the influence of viral activity on berry physiology.

In almost all viral diseases occurring in the vineyard, the virus is distributed systemically throughout the grapevine. Once introduced in the host, viral particles move rapidly within the vascular tissue towards sink tissues and establish infections (Armijo *et al.*, 2016). Although we detected the presence of GRBaV in vegetative and berry tissues during growing and harvest seasons, symptoms of red blotch were only evident after véraison, which suggests that the disease onset is mostly dependent on grapevine phenology and not necessarily linked to viral accumulation. Similar observations have been made during grapevine leafroll disease, supporting the hypothesis that the appearance of viral disease symptoms in the vineyard may result from the interaction between pathogen and host cellular factors at specific phenological stages (Naidu *et al.*, 2014). Whether GRBaV is able to modulate its infection strategy as a function of plant development and/or grapevines have distinct responses to red blotch throughout the season remains to be resolved. GRBaV shares several similarities with geminiviruses, including a small single-stranded DNA genome that encodes six potential proteins (Krenz *et al.*, 2014). Because of their limited protein-coding capacities, geminiviruses rely heavily on host cellular machinery and interact with an assortment of plant proteins and pathways to promote infection (Hanley-Bowdoin *et al.*, 2013). We confirmed the expression of five GRBaV genes in ripening red-skinned berries. Although three of these genes have been assigned putative functions in viral DNA replication and coat formation (Krenz *et al.*, 2014), their specific functions in pathogenesis are yet to be elucidated. Our results indicated that the developmental stage of the berries may not influence the expression of GRBaV viral genes and that host factors could play a more critical role in the establishment of successful infections.

Red blotch symptoms in grape berries include abnormal chemical composition and asynchronous ripening in the clusters (Sudarshana *et al.*, 2015), both reflecting alterations in host metabolic homeostasis and developmental processes. Interestingly, we determined that GRBaV infections induced some processes associated with early fruit development (e.g. photosynthesis, carbon fixation, and inhibition of PME activity) in berries at late stages of ripening, while repressing pathways involved in fruit ripening (e.g. ABA biosynthesis and signaling, secondary metabolic pathways). Abiotic stress responses to hypoxia and temperature were also suppressed by red blotch in grape berries. These observations may imply that the virus, as a biotrophic pathogen, could redirect host metabolic processes to sustain higher energy demands due to viral replication and at the same time suppressing ripening-related events and responses to stress (e.g. oxidative reactions and biosynthesis of secondary metabolites), potentially counterproductive to viral infections. However, further evidence using infected berries from different grape cultivars and environmental conditions will be necessary to understand how both grapevine genotype and environment may influence the disease outcome. It is also important to consider that the effects of GRBaV infections on berries may not be comparable with those occurring in vegetative tissues; for instance, photosynthetic pathways are generally inhibited during viral infections of grape leaves (Sampol *et al.*, 2003; Bertamini *et al.*, 2004).

GRBaV infections restricted the biosynthesis and accumulation of phenylpropanoids and derivatives, which preferentially accumulate after véraison in red-skinned berries. These secondary metabolites function as antioxidants and phytoalexins to protect the berries against a variety of stresses, and are important contributors of berry quality parameters, such as color, flavor, and aroma (Conde *et al.*, 2007; Vogt, 2010). The inhibition of phenylpropanoid metabolism appears to be a hallmark of viral infections in red-skinned berries, as has previously been reported in leafroll-affected berries (Vega *et al.*, 2011). In particular, the anthocyanin biosynthetic pathway was greatly impaired as a result of leafroll and red blotch, correlating with the reduced coloration observed in GRBaV-infected berries from several red-skinned cultivars (Vega *et al.*, 2011; Sudarshana *et al.*, 2015).

In this study, we demonstrated that GRBaV infections compromised the regulation of ripening by: (i) suppressing specific ripening events; (ii) altering the expression patterns of transcription factors that control the transition from the growth to ripening phases (e.g. NAC and MYB families) and the activation of ripening pathways (e.g. AP2-ERFs); and (iii) causing hormonal imbalances. Most of the alterations in the ripening regulatory networks occurred in infected berries at véraison, the same developmental time when red blotch symptoms initiated, suggesting a link between mechanisms involved in the control of grapevine responses to viruses and berry development. Similar observations were made in leafroll-affected berries, which presented lower expression levels of MYB transcription factors at late stages of ripening (Vega *et al.*, 2011).

Plant hormones modulate ripening processes and stress responses in grape berries. Red blotch had a profound impact on ABA, ethylene, and auxin pathways. ABA is considered the triggering signal of berry ripening, since its accumulation coincides with véraison (Kuhn *et al.*, 2014) and ABA-responsive transcription factors have been implicated in the activation of ripening processes (Koyama *et al.*, 2014; Nicolas *et al.*, 2014). Particularly, the induction of anthocyanin biosynthesis in a variety of grape cultivars has been linked to ABA-mediated signaling pathways (Wheeler *et al.*, 2009; Berli *et al.*, 2010; Gambetta *et al.*, 2010). Our results indicate that alterations of ripening events in the GRBaV-infected berries, including the reduced anthocyanin content at late stages of ripening, could be a direct result of lower ABA levels at véraison. ABA has been shown to stimulate ethylene-mediated pathways in berries, and together both hormones appear to regulate the progression of ripening (Chervin *et al.*, 2004; Kuhn *et al.*, 2014). GRBaV infections also affected genes involved in ethylene biosynthesis and responses (e.g. 1-aminocyclopropane-1-carboxylate oxidases and AP2-ERFs), which could further account for the antagonistic effect of red blotch on ripening events and immune responses. In contrast, red blotch promoted auxin-mediated pathways, known to suppress berry ripening. Auxins play crucial roles in the early development of grape berries and are known to inhibit ripening processes by delaying ABA-triggered processes (Gouthu and Deluc, 2015).

The mechanisms by which grapevine viruses cause disease in ripening berries need to be investigated. GRBaV may actively interfere with the regulation of berry ripening by hijacking the plant post-transcriptional control. We identified elements of the host post-transcriptional machinery that were misregulated in GRBaV-infected berries. Post-transcriptional regulation intersects plant immune responses, developmental transitions, and hormone signaling (Vaucheret, 2006). Plant viruses, in particular geminiviruses, encode multiple silencing suppressors that interfere with host siRNA production and alter plant DNA methylation and miRNA pathways, causing developmental defects (Hanley-Bowdoin *et al.*, 2013). A previous study on leafroll indicated that the viral infection caused up-regulation of Dicer-like genes in ripening berries (Vega *et al.*, 2011). Therefore, the relationship between the viral-induced repression of host RNA silencing processes and the post-transcriptional regulation of ripening should be addressed from the perspective of compatible grapevine–virus interactions.

## Supplementary data

Supplementary data are available at *JXB* online.

Fig. S1. Geographic locations of the two vineyards in California where berry sampling was performed.

Fig. S2. Scatterplots showing the correlation between the fold changes (log_2_) in expression obtained by processing the RNAseq data with DESeq2 and the fold changes (log_2_) measured by qRT-PCR.

Fig. S3. Viral titers in grape berries based on qPCR amplification of GRBaV DNA.

Table S1. Weather conditions in the vineyards at the time of sampling.

Table S2. Primer sequences used for qRT-PCR.

Table S3. Maturity indices of the berries used in the study

Table S4. Summary of parsed and mapped reads of mRNA from GRBaV-positive berries and healthy controls.

Table S5. Differential expression of grape genes due to GRBaV infections of berries at four stages of ripening.

Table S6. Enriched grape functional categories (*P*≤0.05) among significantly up- and down-regulated genes in grape berries infected by GRBaV when compared with control berries.

Table S7. Activity of enzymes from the core and peripheral phenylpropanoid pathways in GRBaV-positive and healthy control berries.

Table S8. Abundance of secondary metabolites in GRBaV-positive and healthy control berries measured by a targeted LC-DAD-MS/MS approach.

Table S9. Intersection between ripening-related genes and GRBaV-responsive genes.

## Supplementary Material

Supplementary_Figures_S1_S3

Supplementary_Table_S1

Supplementary_Table_S2

Supplementary_Table_S3

Supplementary_Table_S4

Supplementary_Table_S5

Supplementary_Table_S6

Supplementary_Table_S7

Supplementary_Table_S8

Supplementary_Table_S9

## References

[CIT0001] Al Rwahnih M Rowhani A Golino DA 2015 First report of Grapevine red blotch associated virus in archival grapevine material from Sonoma County, California. Plant Disease 99, 895.

[CIT0002] Albacete A Ghanem ME Martínez-Andújar C Acosta M Sánchez-Bravo J Martínez V Lutts S Dodd IC Pérez-Alfocea F 2008 Hormonal changes in relation to biomass partitioning and shoot growth impairment in salinized tomato (*Solanum lycopersicum* L.) plants. Journal of Experimental Botany 59, 4119–4131. 19036841 10.1093/jxb/ern251 PMC2639025

[CIT0003] Alexandersson E Jacobson D Vivier MA Weckwerth W Andreasson E 2014 Field-omics—understanding large-scale molecular data from field crops. Frontiers in Plant Science 5, 286. 24999347 10.3389/fpls.2014.00286 PMC4064663

[CIT0004] Armijo G Schlechter R Agurto M Muñoz D Nuñez C Arce-Johnson P 2016 Grapevine pathogenic microorganisms: understanding infection strategies and host response scenarios. Frontiers in Plant Science 7, 382. 27066032 10.3389/fpls.2016.00382 PMC4811896

[CIT0005] Atkinson NJ Urwin PE 2012 The interaction of plant biotic and abiotic stresses: from genes to the field. Journal of Experimental Botany 63, 3523–3543. 22467407 10.1093/jxb/ers100

[CIT0006] Berli FJ Moreno D Piccoli P Hespanhol-Viana L Silva MF Bressan-Smith R Cavagnaro JB Bottini R 2010 Abscisic acid is involved in the response of grape (*Vitis vinifera* L.) cv. Malbec leaf tissues to ultraviolet-B radiation by enhancing ultraviolet-absorbing compounds, antioxidant enzymes and membrane sterols. Plant, Cell and Environment 33, 1–10. 10.1111/j.1365-3040.2009.02044.x 19781012

[CIT0007] Bertamini M Muthuchelian K Nedunchezhian N 2004 Effect of grapevine leafroll on the photosynthesis of field grown grapevine plants (*Vitis vinifera* L. cv. Lagrein). Journal of Phytopathology 152, 145–152.

[CIT0008] Blanco-Ulate B Amrine KC Collins TS 2015 Developmental and metabolic plasticity of white-skinned grape berries in response to *Botrytis cinerea* during noble rot. Plant Physiology 169, 2422–2443. 26450706 10.1104/pp.15.00852 PMC4677888

[CIT0009] Blanco-Ulate B Vincenti E Powell AL Cantu D 2013 Tomato transcriptome and mutant analyses suggest a role for plant stress hormones in the interaction between fruit and *Botrytis cinerea*. Frontiers in Plant Science 4, 142. 23717322 10.3389/fpls.2013.00142 PMC3653111

[CIT0010] Bogs J Jaffé FW Takos AM Walker AR Robinson SP 2007 The grapevine transcription factor VvMYBPA1 regulates proanthocyanidin synthesis during fruit development. Plant Physiology 143, 1347–1361. 17208963 10.1104/pp.106.093203 PMC1820911

[CIT0011] Bradford MM 1976 A rapid and sensitive method for the quantitation of microgram quantities of protein utilizing the principle of protein–dye binding. Analytical Biochemistry 72, 248–254. 942051 10.1016/0003-2697(76)90527-3

[CIT0012] Camont L Cottart CH Rhayem Y Nivet-Antoine V Djelidi R Collin F Beaudeux JL Bonnefont-Rousselot D 2009 Simple spectrophotometric assessment of the trans-/cis-resveratrol ratio in aqueous solutions. Analytica Chimica Acta 634, 121–128. 19154820 10.1016/j.aca.2008.12.003

[CIT0013] Cavallini E Matus JT Finezzo L 2015 The phenylpropanoid pathway is controlled at different branches by a set of R2R3-MYB C2 repressors in grapevine. Plant Physiology 167, 1448–1470. 25659381 10.1104/pp.114.256172 PMC4378173

[CIT0014] Chen X Zhang Z Liu D Zhang K Li A Mao L 2010 SQUAMOSA promoter-binding protein-like transcription factors: star players for plant growth and development. Journal of Integrative Plant Biology 52, 946–951. 20977652 10.1111/j.1744-7909.2010.00987.x

[CIT0015] Chervin C El-Kereamy A Roustan J-P Latché A Lamon J Bouzayen M 2004 Ethylene seems required for the berry development and ripening in grape, a non-climacteric fruit. Plant Science 167, 1301–1305.

[CIT0016] Chin C-S Peluso P Sedlazeck FJ 2016 Phased diploid genome assembly with single molecule real-time sequencing. Nature Methods 13, 1050–1054. 27749838 10.1038/nmeth.4035 PMC5503144

[CIT0017] Conde C Silva P Fontes N Dias ACP Tavares RM Sousa MJ Agasse A Delrot S Gerós H 2007 Biochemical changes throughout grape berry development and fruit and wine quality. Food 1, 1–22.

[CIT0018] Coombe BG 1995 Growth stages of the grapevine: adoption of a system for identifying grapevine growth stages. Australian Journal of Grape and Wine Research, 1, 104–110.

[CIT0019] Culver JN Padmanabhan MS 2007 Virus-induced disease: altering host physiology one interaction at a time. Annual Review of Phytopathology 45, 221–243. 10.1146/annurev.phyto.45.062806.094422 17417941

[CIT0020] Davies C Boss PK Gerós H Lecourieux F Delrot S 2012 Source/sink relationships and molecular biology of sugar accumulation in grape berries. In: Gerós H Chaves M Delrot S, eds. The biochemistry of the grape berry, Vol. 1 Dubai, UAE: Bentham Science Publishers, 44–66.

[CIT0021] de Ferrars RM Czank C Saha S Needs PW Zhang Q Raheem KS Botting NP Kroon PA Kay CD 2014 Methods for isolating, identifying, and quantifying anthocyanin metabolites in clinical samples. Analytical Chemistry 86, 10052–10058. 24828315 10.1021/ac500565a

[CIT0022] Deluc L Barrieu F Marchive C Lauvergeat V Decendit A Richard T Carde JP Mérillon JM Hamdi S 2006 Characterization of a grapevine R2R3-MYB transcription factor that regulates the phenylpropanoid pathway. Plant Physiology 140, 499–511. 16384897 10.1104/pp.105.067231 PMC1361319

[CIT0023] Deluc L Bogs J Walker AR Ferrier T Decendit A Merillon JM Robinson SP Barrieu F 2008 The transcription factor VvMYB5b contributes to the regulation of anthocyanin and proanthocyanidin biosynthesis in developing grape berries. Plant Physiology 147, 2041–2053. 18539781 10.1104/pp.108.118919 PMC2492604

[CIT0024] Gambetta GA Matthews MA Shaghasi TH McElrone AJ Castellarin SD 2010 Sugar and abscisic acid signaling orthologs are activated at the onset of ripening in grape. Planta 232, 219–234. 20407788 10.1007/s00425-010-1165-2 PMC2872022

[CIT0025] Gouthu S Deluc LG 2015 Timing of ripening initiation in grape berries and its relationship to seed content and pericarp auxin levels. BMC Plant Biology 15, 46. 25848949 10.1186/s12870-015-0440-6 PMC4340107

[CIT0026] Grimplet J Cramer GR Dickerson JA Mathiason K Van Hemert J Fennell AY 2009 VitisNet: ‘Omics’ integration through grapevine molecular networks. PLoS One 4, e8365. 20027228 10.1371/journal.pone.0008365 PMC2791446

[CIT0027] Gutterson N Reuber TL 2004 Regulation of disease resistance pathways by AP2/ERF transcription factors. Current Opinion in Plant Biology 7, 465–471. 15231271 10.1016/j.pbi.2004.04.007

[CIT0028] Hanley-Bowdoin L Bejarano ER Robertson D Mansoor S 2013 Geminiviruses: masters at redirecting and reprogramming plant processes. Nature Reviews. Microbiology 11, 777–788. 24100361 10.1038/nrmicro3117

[CIT0029] Höll J Vannozzi A Czemmel S 2013 The R2R3-MYB transcription factors MYB14 and MYB15 regulate stilbene biosynthesis in *Vitis vinifera*. The Plant Cell 25, 4135–4149. 24151295 10.1105/tpc.113.117127 PMC3877794

[CIT0030] Karlova R Chapman N David K Angenent GC Seymour GB de Maagd RA 2014 Transcriptional control of fleshy fruit development and ripening. Journal of Experimental Botany 65, 4527–4541. 25080453 10.1093/jxb/eru316

[CIT0031] Kodan A Kuroda H Sakai F 2002 A stilbene synthase from Japanese red pine (Pinus densiflora): implications for phytoalexin accumulation and down-regulation of flavonoid biosynthesis. Proceedings of the National Academy of Sciences, USA 99, 3335–3339. 10.1073/pnas.042698899 PMC122519 11880657

[CIT0032] Kou X Wang S Wu M Guo R Xue Z Meng N Tao X Chen M Zhang Y 2014 Molecular characterization and expression analysis of NAC family transcription factors in tomato. Plant Molecular Biology Reporter 32, 501–516.

[CIT0033] Koyama K Numata M Nakajima I Goto-Yamamoto N Matsumura H Tanaka N 2014 Functional characterization of a new grapevine MYB transcription factor and regulation of proanthocyanidin biosynthesis in grapes. Journal of Experimental Botany 65, 4433–4449. 24860184 10.1093/jxb/eru213

[CIT0034] Krenz B Thompson JR Fuchs M Perry KL 2012 Complete genome sequence of a new circular DNA virus from grapevine. Journal of Virology 86, 7715. 22733880 10.1128/JVI.00943-12 PMC3416304

[CIT0035] Krenz B Thompson JR McLane HL Fuchs M Perry KL 2014 Grapevine red blotch-associated virus is widespread in the United States. Phytopathology 104, 1232–1240. 24805072 10.1094/PHYTO-02-14-0053-R

[CIT0036] Kuhn N Guan L Dai ZW 2014 Berry ripening: recently heard through the grapevine. Journal of Experimental Botany 65, 4543–4559. 24285825 10.1093/jxb/ert395

[CIT0037] Laliberté JF Sanfaçon H 2010 Cellular remodeling during plant virus infection. Annual Review of Phytopathology 48, 69–91. 10.1146/annurev-phyto-073009-114239 20337516

[CIT0038] Licausi F Giorgi FM Zenoni S Osti F Pezzotti M Perata P 2010 Genomic and transcriptomic analysis of the AP2/ERF superfamily in *Vitis vinifera*. BMC Genomics 11, 719. 21171999 10.1186/1471-2164-11-719 PMC3022922

[CIT0039] Licausi F Ohme-Takagi M Perata P 2013 APETALA2/Ethylene Responsive Factor (AP2/ERF) transcription factors: mediators of stress responses and developmental programs. New Phytologist 199, 639–649. 24010138 10.1111/nph.12291

[CIT0040] Lionetti V Raiola A Mattei B Bellincampi D 2015 The grapevine *VvPMEI1* gene encodes a novel functional pectin methylesterase inhibitor associated to grape berry development. PLoS One 10, e0133810. 26204516 10.1371/journal.pone.0133810 PMC4512722

[CIT0041] Love MI Huber W Anders S 2014 Moderated estimation of fold change and dispersion for RNA-seq data with DESeq2. Genome Biology 15, 550. 25516281 10.1186/s13059-014-0550-8 PMC4302049

[CIT0042] Ma K Xiao J Li X Zhang Q Lian X 2009 Sequence and expression analysis of the C3HC4-type RING finger gene family in rice. Gene 444, 33–45. 19523506 10.1016/j.gene.2009.05.018

[CIT0043] Maliogka VI Martelli GP Fuchs M Katis NI 2015 Control of viruses infecting grapevine. Advances in Virus Research 91, 175–227. 25591880 10.1016/bs.aivir.2014.11.002

[CIT0044] Martelli GP 2014 Directory of virus and virus-like diseases of the grapevine and their agents. Journal of Plant Pathology 96, 1–136.

[CIT0045] Matus JT Aquea F Arce-Johnson P 2008 Analysis of the grape MYB R2R3 subfamily reveals expanded wine quality-related clades and conserved gene structure organization across Vitis and Arabidopsis genomes. BMC Plant Biology 8, 83. 18647406 10.1186/1471-2229-8-83 PMC2507771

[CIT0046] Naidu R Rowhani A Fuchs M Golino D Martelli GP 2014 Grapevine leafroll: a complex viral disease affecting a high-value fruit crop. Plant Disease 98, 1172–1185. 10.1094/PDIS-08-13-0880-FE 30699617

[CIT0047] Nakashima K Takasaki H Mizoi J Shinozaki K Yamaguchi-Shinozaki K 2012 NAC transcription factors in plant abiotic stress responses. Biochimica et Biophysica Acta 1819, 97–103. 22037288 10.1016/j.bbagrm.2011.10.005

[CIT0048] Nelson RS Citovsky V 2005 Plant viruses. Invaders of cells and pirates of cellular pathways. Plant Physiology 138, 1809–1814. 16172093 10.1104/pp.104.900167 PMC1183372

[CIT0049] Nicolas P Lecourieux D Kappel C Cluzet S Cramer G Delrot S Lecourieux F 2014 The basic leucine zipper transcription factor ABSCISIC ACID RESPONSE ELEMENT-BINDING FACTOR2 is an important transcriptional regulator of abscisic acid-dependent grape berry ripening processes. Plant Physiology 164, 365–383. 24276949 10.1104/pp.113.231977 PMC3875815

[CIT0050] Pallas V García JA 2011 How do plant viruses induce disease? Interactions and interference with host components. Journal of General Virology 92, 2691–2705. 21900418 10.1099/vir.0.034603-0

[CIT0051] Palumbo MC Zenoni S Fasoli M Massonnet M Farina L Castiglione F Pezzotti M Paci P 2014 Integrated network analysis identifies fight-club nodes as a class of hubs encompassing key putative switch genes that induce major transcriptome reprogramming during grapevine development. The Plant Cell 26, 4617–4635. 25490918 10.1105/tpc.114.133710 PMC4311215

[CIT0052] Pilati S Perazzolli M Malossini A 2007 Genome-wide transcriptional analysis of grapevine berry ripening reveals a set of genes similarly modulated during three seasons and the occurrence of an oxidative burst at vèraison. BMC Genomics 8, 428. 18034875 10.1186/1471-2164-8-428 PMC2228314

[CIT0053] Podzimska-Sroka D O’Shea C Gregersen PL Skriver K 2015 NAC transcription factors in senescence: from molecular structure to function in crops. Plants 4, 412–448. 27135336 10.3390/plants4030412 PMC4844398

[CIT0054] Poojari S Alabi OJ Fofanov VY Naidu RA 2013 A leafhopper-transmissible DNA virus with novel evolutionary lineage in the family geminiviridae implicated in grapevine redleaf disease by next-generation sequencing. PLoS One 8, e64194. 23755117 10.1371/journal.pone.0064194 PMC3673993

[CIT0055] Sampol B Bota J Riera D Medrano H Flexas J 2003 Analysis of the virus-induced inhibition of photosynthesis in malmsey grapevines. New Phytologist 160, 403–412. 10.1046/j.1469-8137.2003.00882.x 33832175

[CIT0056] Seguin J Rajeswaran R Malpica-López N Martin RR Kasschau K Dolja VV Otten P Farinelli L Pooggin MM 2014 *De novo* reconstruction of consensus master genomes of plant RNA and DNA viruses from siRNAs. PLoS One 9, e88513. 24523907 10.1371/journal.pone.0088513 PMC3921208

[CIT0057] Singh R Rastogi S Dwivedi UN 2010 Phenylpropanoid metabolism in ripening fruits. Comprehensive Reviews in Food Science and Food Safety 9, 398–416. 10.1111/j.1541-4337.2010.00116.x 33467837

[CIT0058] Sudarshana MR Perry KL Fuchs MF 2015 Grapevine red blotch-associated virus, an emerging threat to the grapevine industry. Phytopathology 105, 1026–1032. 25738551 10.1094/PHYTO-12-14-0369-FI

[CIT0059] Suzuki N Rivero RM Shulaev V Blumwald E Mittler R 2014 Abiotic and biotic stress combinations. New Phytologist 203, 32–43. 24720847 10.1111/nph.12797

[CIT0060] Vaucheret H 2006 Post-transcriptional small RNA pathways in plants: mechanisms and regulations. Genes and Development 20, 759–771. 16600909 10.1101/gad.1410506

[CIT0061] Vega A Gutiérrez RA Peña-Neira A Cramer GR Arce-Johnson P 2011 Compatible GLRaV-3 viral infections affect berry ripening decreasing sugar accumulation and anthocyanin biosynthesis in Vitis vinifera. Plant Molecular Biology 77, 261–274. 21786204 10.1007/s11103-011-9807-8

[CIT0062] Vogt T 2010 Phenylpropanoid biosynthesis. Molecular Plant 3, 2–20. 20035037 10.1093/mp/ssp106

[CIT0063] von Arnim AG Deng XW 1993 Ring finger motif of Arabidopsis thaliana COP1 defines a new class of zinc-binding domain. Journal of Biological Chemistry 268, 19626–19631. 8366106

[CIT0064] Walker AR Lee E Bogs J McDavid DA Thomas MR Robinson SP 2007 White grapes arose through the mutation of two similar and adjacent regulatory genes. The Plant Journal 49, 772–785. 17316172 10.1111/j.1365-313X.2006.02997.x

[CIT0065] Wheeler S Loveys B Ford C Davies C 2009 The relationship between the expression of abscisic acid biosynthesis genes, accumulation of abscisic acid and the promotion of *Vitis vinifera* L. berry ripening by abscisic acid. Australian Journal of Grape and Wine Research 15, 195–204.

[CIT0066] Wu W Cheng Z Liu M Yang X Qiu D 2014 C3HC4-type RING finger protein NbZFP1 is involved in growth and fruit development in Nicotiana benthamiana. PLoS One 9, e99352. 24901716 10.1371/journal.pone.0099352 PMC4047095

