# Supplementary material for: Red blotch disease alters grape berry development and metabolism by interfering with the transcriptional and hormonal regulation of ripening
Source: J Exp Bot. 2017 Jan 27;68(5):1225–38. doi: 10.1093/jxb/erw506 (PMC5444480; doi:10.1093/jxb/erw506)
Supplement: Supplementary_Figures_S1_S3 [file erw506_suppl_Supplementary_Figures_S1_S3.pdf]

Red blotch disease alters grape berry development and metabolism by interfering with the transcriptional and hormonal regulation of ripening

Barbara Blanco-Ulate, Helene Hopfer, Rosa Figueroa-Balderas, Zirou Ye, Rosa M. Rivero, Alfonso Albacete, Francisco Pérez-Alfocea, Renata Koyama, Michael M. Anderson, Rhonda J. Smith, Susan E. Ebeler, and Dario Cantu

Supplementary Figures

Figure S1. Geographic location of the two vineyards in California where berry sampling was performed.

Figure S2. Scatterplots showing the correlation between the fold changes (log2) in expression obtained by processing the RNAseq data with DESeq2 and the fold changes (log2) measured by qRT-PCR. Relative expression was calculated using VIT_02s0012g00910, VIT_18s0001g00360 and VIT_04s0044g00580 (VvActin to choose the best performing reference gene. Linear trends, correlation coefficients (r) and P-values are provided.

Figure S3. Viral titers in grape berries based on qPCR amplification of GRBaV DNA. Pre-v, Pre-véraison; V, véraison; Post-v, post-véraison; H, harvest. Error bars correspond to the standard error.

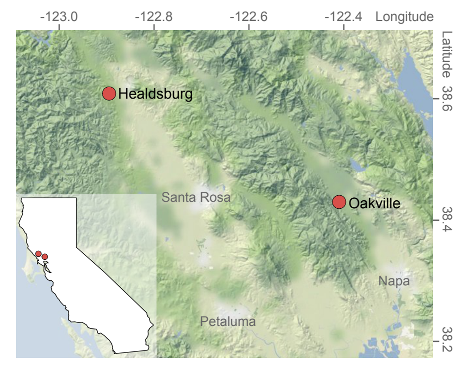
-123.0
-122.8
-122.6
-122.4
Longitude
Latitude
38.6
38.4
38.2
Healdsburg
Oakville
Santa Rosa
Napa
Petaluma

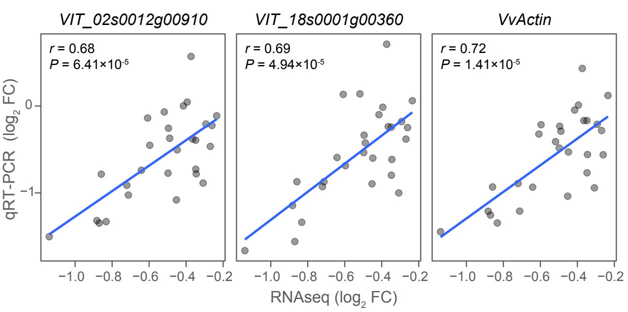
VIT_02s0012g00910
r = 0.68
P = 6.41×10-5
VIT_18s0001g00360
r = 0.69
P = 4.94×10-5
VvActin
r = 0.72
P = 1.41×10-5
qRT-PCR (log2 FC)
0
−1
−1.0
−0.8
−0.6
−0.4
−0.2
RNAseq (log2 FC)

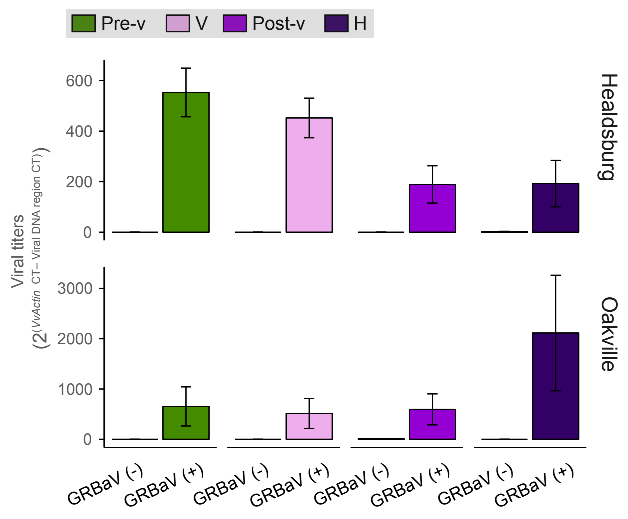

Pre-v
V
Post-v
H
Viral titers
Healdsburg
Oakville
600
400
200
0
3000
2000
1000
0
GRBaV (-)
GRBaV (+)
